# Roles of the Transcription Factors Sfl2 and Efg1 in White-Opaque Switching in a/α Strains of Candida albicans

**DOI:** 10.1128/mSphere.00703-18

**Published:** 2019-04-17

**Authors:** Yang-Nim Park, Kayla Conway, Thomas P. Conway, Karla J. Daniels, David R. Soll

**Affiliations:** a Developmental Studies Hybridoma Bank, Department of Biology, The University of Iowa, Iowa City, Iowa, USA; Carnegie Mellon University

**Keywords:** Candida albicans, Efg1, Sfl2, white-opaque switching

## Abstract

Candida albicans remains the most pervasive fungal pathogen colonizing humans. The majority of isolates from hosts are heterozygous at the mating type locus (*MTL***a**/α), and a third of these have recently been shown to be capable of switching to the opaque phenotype. Here we have investigated the roles of two transcription factors (TFs) Sfl2 and Efg1, in repressing switching in **a**/α strains. Deleting either gene results in the capacity of **a**/α cells to switch to opaque en masse under facilitating environmental conditions, which include *N*-acetylglucosamine (GlcNAc) as the carbon source, physiological temperature (37°C), and high CO_2_ (5%). These conditions are similar to those in the host. Our results further reveal that while glucose is a repressor of *sfl2*Δ and *efg1*Δ switching, GlcNAc is an inducer. Finally, we show that when GlcNAc is the carbon source, and the temperature is low (25°C), the *efg1*Δ mutants, but not the *sfl2*Δ mutants, form a tiny, elongate cell, which differentiates into an opaque cell when transferred to conditions optimal for **a**/α switching. These results demonstrate that at least two TFs, Sfl2 and Efg1, repress switching in **a**/α cells and that **a**/α strains with either an *sfl2*Δ or *efg1*Δ mutation can switch en masse but only under physiological conditions. The role of opaque **a**/α cells in commensalism and pathogenesis must, therefore, be investigated.

**IMPORTANCE** More than 95% of Candida albicans strains isolated from humans are *MTL***a**/α, and approximately a third of these can undergo the white-to-opaque transition. Therefore, besides being a requirement for *MTL*-homozygous strains to mate, the opaque phenotype very likely plays a role in the commensalism and pathogenesis of nonmating, **a**/α populations colonizing humans.

## Abstract (precis)

More than 95% of Candida albicans strains isolated from humans are *MTL***a**/α, and approximately a third of these can undergo the white-to-opaque transition. Therefore, besides being a requirement for *MTL*-homozygous strains to mate, the opaque phenotype very likely plays a role in the commensalism and pathogenesis of nonmating, **a**/α populations colonizing humans.

## INTRODUCTION

Candida albicans remains the most pervasive opportunistic fungal pathogen colonizing humans (1–3). The majority of natural strains are heterozygous (**a**/α) at the mating type locus (4–7) and cannot mate (8, 9). To mate, these cells must undergo homozygosis at the mating type locus (4, 8–11) and then switch from the yeast phase “white” phenotype to the unique “opaque” phase phenotype (10, 11). Thus, a paradigm was established that only *MTL*-homozygous strains (**a/a** and α/α) could switch to opaque and that the opaque phase cell served as the mating-competent phenotype. This paradigm was altered in 2013 when Xie et al. (7) found that approximately one third of a collection of **a**/α strains isolated from hosts underwent white-opaque switching, forming mating-incompetent opaque cells, albeit at low frequencies, when cultured on medium with *N*-acetylglucosamine (GlcNAc) at 25°C in 5% CO_2_ (7). These results suggested that in a significant minority of colonized hosts, the **a**/α strain forms opaque cells that play a role in colonization. Here, we have investigated the roles of two transcription factors, Sfl2 and Efg1, in **a**/α switching, by using deletion mutants generated in two **a**/α strains that did not switch. *SFL2* is upregulated in cells during growth at 37°C, but not at lower temperatures, and is involved in *EFG1*-dependent regulation of hypha formation (12, 13). Efg1 has also been shown to be a repressor of white-opaque switching in C. albicans (14–16). Xie et al. (7) previously provided evidence that Efg1 was a repressor of switching in **a**/α strains. Here, parent **a**/α strains and *sfl2Δ*, *efg1Δ*, and *sfl2Δ efg1Δ* mutants, were tested for switching under eight sets of conditions that included all combinatorial permutations of sugar source (glucose versus GlcNAc), temperature (25°C versus 37°C), and CO_2_ level (0.04% [air] versus 5%).

Our results indicate the following. (i) **a**/α *sfl2*Δ and *efg1Δ* mutants and the *sfl2*Δ *efg1*Δ double mutant undergoes mass conversion (>90%) from white to opaque on GlcNAc-containing agar at 37°C in 5% CO_2_. (ii) The effects of environmental conditions on white-to-opaque switching by the *sfl2*Δ and *efg1*Δ mutants are similar. (iii) *WOR1*, *OP4*, and a number of other genes associated with switching are upregulated in opaque cells of both mutants. (iv) The stability of the opaque phenotype of the two mutants differ at 25°C. (v) Glucose is an inhibitor and GlcNAc is an inducer of switching. (vi) *efg1*Δ cells, but not **a**/α *sfl2*Δ cells, form tiny, elongate cells on GlcNAc agar at 25°C. (vii) These tiny, elongate cells, when incubated under optimal conditions for switching, grow directly into opaque cells. The observations that approximately a third of natural **a**/α C. albicans strains switch and that switching en masse by **a**/α mutants of repressor genes requires all three physiological conditions suggest that the opaque phenotype may be expressed in a third of C. albicans infections. Further investigation of **a**/α switching and the role of **a**/α opaque cells in pathogenesis is therefore warranted.

## RESULTS

### The two parent a/α strains do not undergo white-to-opaque switching.

**FIG 1:**
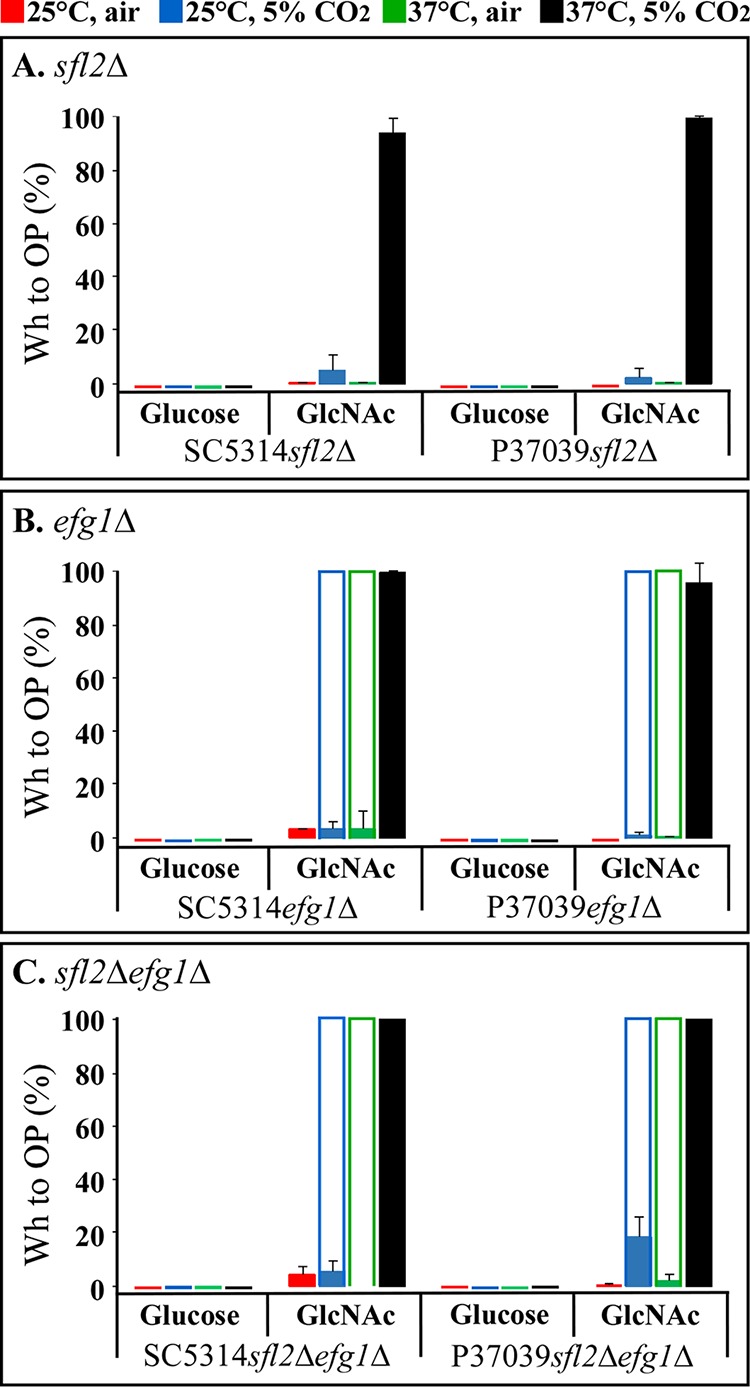
White-to-opaque switching by *sfl2*Δ, *efg1*Δ, and *sfl2*Δ *efg1*Δ mutants generated in the **a**/α wild-type strains SC5314 and P37039 on glucose or GlcNAc agar under four sets of environmental conditions. The sets of conditions are presented at the top of the figure. (A) *sfl2*Δ mutants; (B) *efg1Δ* mutants; (C) *sfl2*Δ *efg1Δ* mutants. The frequencies of uniformly opaque or opaque-sectored colonies are presented as solid colored bars. Colonies containing a mixture of either yeast, tiny elongate and opaque cells (25°C), or yeast and opaque cells (37°C) are presented as white bars outlined in color. The error bars represent standard deviations for data from at least three experiments. The quantitative data for these bar graphs are presented in Table 1.

**FIG 2:**
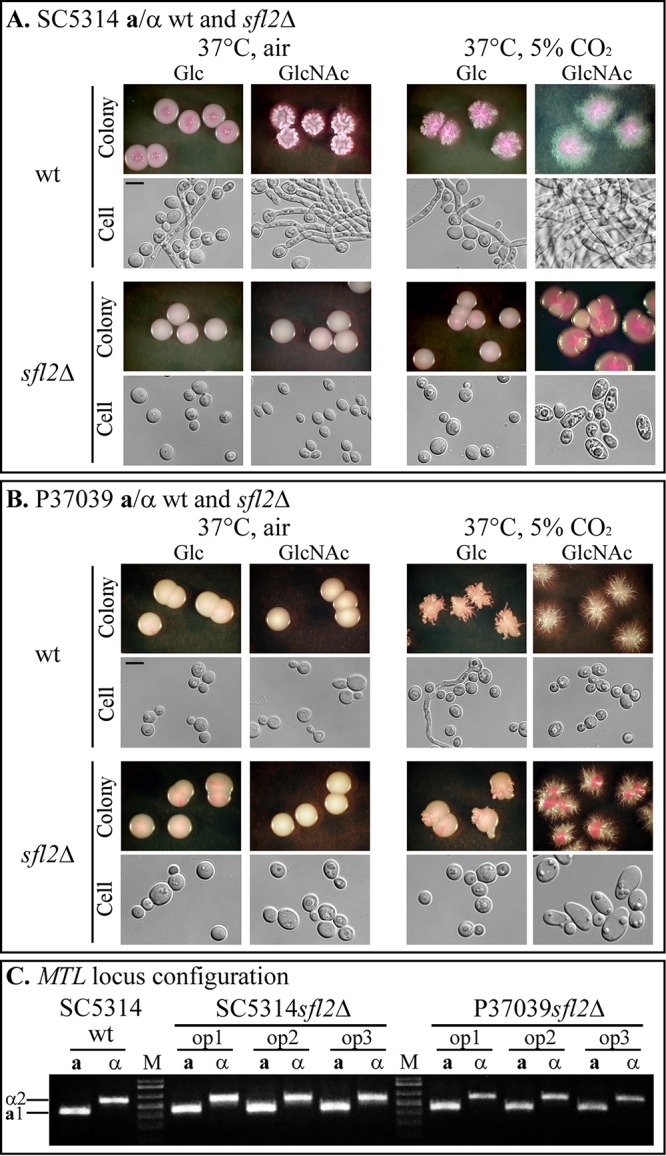
Colony and cell phenotypes of the **a**/α parental wild-type and *sfl2*Δ mutants on glucose or GlcNAc agar at 37°C in air or 5% CO_2_ and maintenance by opaque cells of the **a**/α configuration of the *MTL* locus. (A) **a**/α SC5314 and the *sfl2Δ* mutant; (B) **a**/α P37039 and the *sfl2Δ* mutant. wt, wild type; Glc, glucose. Bars, 5 µm. (C) Configuration of the *MTL* locus of the **a**/α SC5314 parental strain, three clonal opaque colonies of **a**/α SC5314*sfl2Δ*, and clonal opaque colonies of **a**/α P37039*sfl2*Δ.

Exploring the roles of Sfl2 and Efg1 in repressing switching by mutant analysis required parental **a**/α strains that did not switch. We selected two wild-type (wt) **a**/α strains, SC5314 (17) and P37039 (18), which in preliminary studies did not switch from white to opaque under the conditions employed by Xie et al. (7). The two strains were tested for switching under eight sets of conditions, which included all combinatorial permutations of three environmental parameters, carbon source (1.25% glucose versus 2% GlcNAc), temperature (25°C versus 37°C), and CO_2_ level (air [0.04% CO_2_] versus 5% CO_2_). The frequency of white-to-opaque switching was assessed at the colony level on supplemented Lee’s agar (19) containing either 1.25% glucose or 2% GlcNAc as the carbon source. “Opaque colonies” were assessed as those fully opaque or with one or more opaque sectors. Data are means ± standard deviations from three or more independent experiments. Neither of the two **a**/α parent strains switched from white to opaque under any of the eight sets of conditions (Fig. 1A and Table 1). Regardless of colony morphologies under the various sets of conditions, no opaque cells were observed microscopically (Fig. 2A and B, respectively).

**TABLE 1:** White-to-opaque switching frequencies of the **a**/α wild-type SC5314 and P37039 strains and their *sfl2*Δ, *efg1*Δ, and *sfl2*Δ *efg1*Δ mutants^*a*^

Strain	Carbon source	25°C, air		25°C, 5% CO_2_		37°C, air		37°C, 5% CO_2_	
		Total col. no.^*b*^	Frequency (%)^*c*^	Total col. no.	Frequency (%)	Total col. no.	Frequency (%)	Total col. no.	Frequency (%)
SC5314 wt	Glucose	1,278	0	1,298	0	1,287	0	1,441	0
	GlcNAc	1,458	0	1,251	0	1,135	0	1,401	0
SC5314 *sfl2*Δ	Glucose	3,797	0	2,033	0	3,015	0	2,740	0
	GlcNAc	2,101	0.1 ± 0.2	3,701	4.9 ± 5.7	3,298	0.0 ± 0.1	2,544	93.8 ± 5.8
SC5314 *efg1*Δ	Glucose	2,986	0	1,696	0	2,166	0	2,828	0
	GlcNAc	1,567	3.1 ± 3.3^*d*^	1,467	3.3 ± 2.6^*e*^	2,235	3.4 ± 6.8^*f*^	2,341	100
SC5314 *sfl2*Δ*efg1*Δ	Glucose	3,527	0	2,214	0	2,825	0	2,826	0
	GlcNAc	2,209	4.5 ± 2.4^*d*^	2,511	5.6 ± 4.0^*e*^	2,698	0^*f*^	2,678	100

P37039 wt	Glucose	1,464	0	1,201	0	1,299	0	1,315	0
	GlcNAc	1,330	0	1,178	0	1,156	0	1,370	0
P37039 *sfl2*Δ	Glucose	2,618	0	1,734	0	2,038	0	2,128	0
	GlcNAc	1,969	0	2,837	1.9 ± 3.6	1,763	0	2,243	99.6 ± 0.6
P37039 *efg1*Δ	Glucose	2,315	0	1,158	0	1,299	0	1,315	0
	GlcNAc	1,354	0^*d*^	1,962	1.0 ± 1.0^*e*^	1,480	0.2 ± 0.3^*f*^	1,849	95.8 ± 7.2
P37039 *sfl2*Δ*efg1*Δ	Glucose	2,195	0	1,167	0	1,615	0	1,739	0
	GlcNAc	1,141	0.3 ± 0.4^*d*^	2,421	18.3 ± 7.5^*e*^	1,242	1.9 ± 2.4^*f*^	1,578	100

a Total colony numbers and white-to-opaque switching frequencies of **a**/α wild-type (wt) SC5314 and P37039 strains and their *sfl2*Δ, *efg1*Δ, and *sfl2*Δ *efg1*Δ mutants grown under four different conditions on two different carbon sources. For the *efg1*Δ mutants, the formation of “white” colonies containing tiny, elongate cells or mixtures of tiny, elongate and opaque cells is noted by footnotes *d* to *f*.

b Total col. no., total colony number.

c The frequencies are presented as the means ± standard deviations for three or more separate experiments.

d Colonies assessed as “white” were composed of a majority of tiny, elongate cells and a small minority of round yeast cells (Fig. 5B).

e Colonies assessed as “white” were composed of a mixture of tiny, elongate cells and opaque cells, varying in proportions (Fig. 5B).

f Colonies assessed as “white” were composed of a majority of opaque cells and very minor proportions of white yeast phase cells (Fig. 5B).

### White-to-opaque switching by a/α *sfl2Δ* mutants.

**FIG 3:**
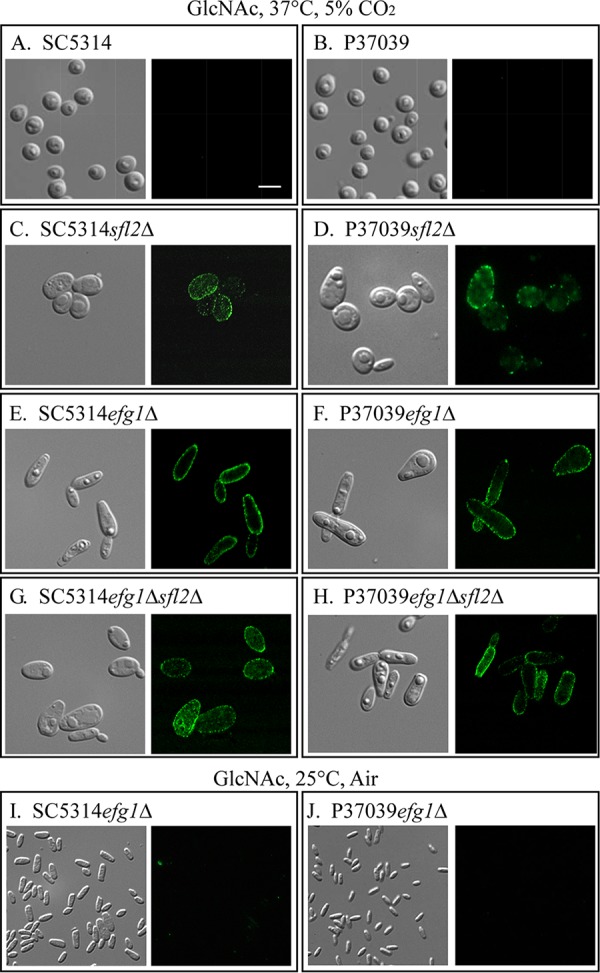
Immunostaining of opaque-cell-specific pimples. Cells were immunostained with antipimple antiserum and an Alexa Fluor 488-tagged secondary anti-rabbit IgG antibody. (A and B) White cells of the parental **a**/α strains SC5314 (A) and P37039 (B). Bar in panel A, 2 μm. (C and D) Opaque cells of the *sfl2Δ* mutants. (E and F) Opaque cells of the *efg1Δ* mutants. (G and H) Opaque cells of the *efg1Δ sfl2Δ* mutants. (I and J) Tiny, elongate cells of *efg1Δ* mutants. Opaque and white cells were obtained from opaque and white colonies, respectively, anf grown on GlcNAc agar at 37°C in 5% CO_2_. Tiny elongate cells were obtained from GlcNAc agar cultures grown at 25°C in air. The latter cells were suspended in medium, the larger opaque cells were allowed to settle, and the supernatant, enriched for tiny cells, was imaged.

Both alleles of *SFL2* were deleted in each of the two wild-type **a**/α strains to generate SC5314*sfl2*Δ and P37039*sfl2*Δ mutants (see Table S1 in the supplemental material). With glucose as the carbon source, neither SC5314*sfl2*Δ nor P37039*sfl2*Δ switched to opaque under any of the four sets of conditions (25°C, air; 25°C, 5% CO_2_; 37°C, air; 37°C, 5% CO_2_) (Table 1 and Fig. 1A), as was the case for the parental strains (Table 1). With GlcNAc as the carbon source, both *sfl2*Δ mutants SC5314*sfl2*Δ and P37039*sfl2*Δ switched at low to negligible frequencies at 25°C in air (0.1 and 0%, respectively) and at low frequencies at 25°C in 5% CO_2_, (4.9 and 1.9%, respectively) (Table 1 and Fig. 1A). On GlcNAc-containing agar at 37°C in air, the two *sfl2*Δ mutants also switched at low to negligible frequencies, but at 37°C in 5% CO_2_, both switched from white to opaque en masse (94% and 100%, respectively) (Fig. 1A and Table 1). The opaque colonies and the opaque sectors contained cells with signature opaque morphologies (20, 21) (Fig. 2A and B). White colonies, regardless of colony morphology (round versus irregular with filamentous edge), contained typical round white cells (Fig. 2A and B), or typical white cells and hyphae. The surfaces of mature opaque cells selectively stained in a punctate fashion with the opaque cell-specific, antipimple polyclonal antibody (22) (Fig. 3C and D, respectively). PCR analysis revealed that opaque cells remained **a**/α (Fig. 2C). These results indicate that mass conversion of the *sfl2*Δ mutants from white to opaque occurs only on GlcNAc-containing agar at 37°C in 5% CO_2_.

10.1128/mSphere.00703-18.2 TABLE S1 Strains used in this study. The two independently generated strains from the wild-type **a**/α strains SC5314 and P37039 were indicated by the letters A and B after the strain names. Download Table S1, DOCX file, 0.02 MB. Copyright © 2019 Park et al. 2019 Park et al. This content is distributed under the terms of the Creative Commons Attribution 4.0 International license.

### a/α *sfl2*Δ opaque cells do not mate.

Xie and coworkers (7) found that opaque cells formed by natural **a**/α strains were incapable of mating. To test whether opaque **a**/α *sfl2*Δ cells mated, we generated an **a**/α SC5314*sfl2*Δ derivative, SC5314*sfl2*Δ-Hr and an **a**/α P37039*sfl2*Δ derivative, P37039*sfl2*Δ-Hr, both resistant to hygromycin B (Table S1). We also generated the *MTL*-homozygous mating partners P37005-Sr (**a/a**) and WO-1-Sr (α/α), both resistant to nourseothricin. A hygromycin B-resistant **a/a** strain, P37005-Hr (**a/a**), was also generated to measure the mating competency of the *MTL*-homozygous mating partners (Table 2). Combined resistance indicated mating. Control crosses of opaque **a/a** and α/α cells resulted in frequencies of mating of 4 × 10^−3^ and 6.7 × 10^−3^ after 2 and 4 days of incubation, respectively (Table 2). Crosses of opaque **a**/α and **a/a** cells resulted in frequencies of 2.8 × 10^−7^ to <8.7 × 10^−9^ (Table 2). Crosses of opaque **a**/α and α/α cells resulted in frequencies of 3.5 × 10^−7^ to <9.5 × 10^−9^ (Table 2). The frequencies of hygromycin B/nourseothricin-resistant offspring resulting from the **a**/α *sfl2*Δ × **a/a** or **a**/α *sfl2*Δ × α/α opaque cell crosses were more than 5 orders of magnitude lower than that of **a**/**a** × α/α opaque cell crosses, indicating that *MTL*-heterozygous *sfl2*Δ opaque cells did not acquire the capacity to mate.

**TABLE 2:** Opaque cells of **a**/α *sfl2Δ* mutants do not mate^*a*^

Strains crossed^*b*^	Mating period (no. of days)	Mating frequency^*c*^
P37005-Hr (**a/a**) × WO-1-Sr (α/α)	2	(4.0 ± 0.2) × 10^−3^
	4	(6.7 ± 0.4) × 10^−3^
P37005-Sr (**a/a**) × SC5314*sfl2*Δ-Hr (**a**/α)	2	(<1.3 ± 0.1) × 10^−8^
	4	(2.8 ± 3.5) × 10^−7^
P37005-Sr (**a/a**) × P37039*sfl2*Δ-Hr (**a**/α)	2	(<1.3 ± 0.2) × 10^−8^
	4	(<8.7 ± 1.6) × 10^−9^

WO-1-Sr (α/α) × SC5314*sfl2Δ*-Hr (**a**/α)	2	(<1.1 ± 0.1) × 10^−8^
	4	(3.5 ± 2.3) × 10^−7^
WO-1-Sr (α/α) × P37039*sfl2Δ*-Hr (**a**/α)	2	(<1.1 ± 0.1) × 10^−8^
	4	(<9.5 ± 2.0) × 10^−9^

a Mating was tested on GlcNAc agar at 25°C in air. Mating was assessed as the frequencies of offspring in the population resistant to both hygromycin B and nourseothricin. Descriptions of the strains generated for mating are presented in Table S1 in the supplemental material. At the end of the strain names, Hr and Sr stand for hygromycin B resistant and nourseothricin resistant, respectively.

b All cells tested were in the opaque phase.

c Frequencies are presented as means ± standard deviations.

### Dependence of a/α *sfl2*Δ switching on *WOR1*.

For *MTL*-hemizygous or *MTL*-homozygous cells, switching depends on the expression of *WOR1* (also referred to as *TOS9*) (23–25). Xie et al. (7) also showed by mutational analyses that **a**/α switching depended on *WOR1*. To test whether white-to-opaque switching in **a**/α *sfl2*Δ mutants required *WOR1*, we deleted *WOR1*, generating strains SC5314*sfl2*Δ*wor1*Δ and P37039*sfl2*Δ*wor1*Δ (Table S1). No switching was observed in either of the **a**/α *sfl2Δ wor1*Δ mutants under any of the eight sets of conditions (Table S3). These results demonstrate that white-to-opaque switching by the **a**/α *sfl2*Δ mutants requires *WOR1*, just as switching by *MTL*-hemizygous and *MTL*-homozygous strains requires *WOR1* (23–25).

### Gene expression in *sfl2*Δ opaque cells.

**FIG 4:**
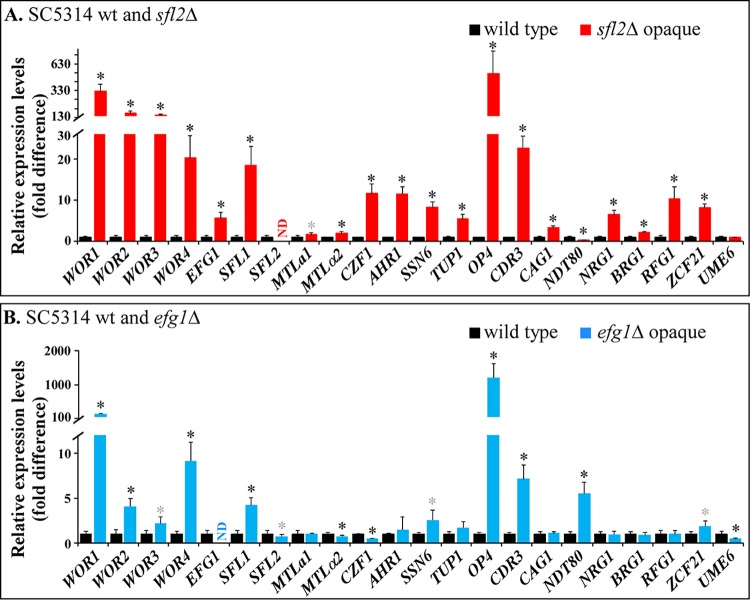
Expression levels of genes associated with switching, in opaque phase cells of **a**/α *sfl2Δ* and *efg1Δ* mutants. The qRT-PCR result for each gene is presented as the relative expression level of cells in mutant colonies versus cells of the parental wild-type **a**/α SC5314 colonies grown on GlcNAc agar at 37°C in 5% CO_2_. While the mutant colonies were opaque, the wild-type colonies were irregular and contained hyphae. (A) SC5314*sfl2*Δ; (B) SC5314*efg1Δ*. Error bars represent standard deviations from three repeats for two separate experiments. wt, wild type; ND, not detectable. Statistical significance compared to the wild type was determined using Student’s *t* test analysis and indicated as follows: gray asterisk, 0.01 < *P* < 0.05; black asterisk, 1 × 10^−13^ < *P <* 0.001.

Expression of *WOR1* and 21 additional genes, including several associated with switching of *MTL*-homozygous strains was compared between wild-type **a**/α SC5314 and *sfl2*Δ cells grown on GlcNAc-containing agar at 37°C in 5% CO_2_. Under these conditions, *sfl2*Δ cell populations exhibited the opaque cell morphology, and more than 95% formed opaque colonies, while wild-type SC5314 cell populations formed colonies containing hyphae (Fig. 2A). Expression of the 21 genes was assessed by qRT-PCR. Relative expression in the *sfl2*Δ mutant was computed as the fold increase or decrease relative to the level of expression in parental SC5314 cells grown under the same conditions. As is evident in Fig. 4A, the level of *WOR1* expression in opaque cells of the **a**/α SC5314*sfl2*Δ strain was 320-fold greater than in **a**/α SC5314 wild-type cells. Expression of the transcription factors (TFs) encoded by *WOR2*, *WOR3*, *WOR4*, *CZF1*, and *AHR1*, all implicated in a network of interacting TFs (26–29), were respectively upregulated approximately 70-fold, 48-fold, 20-fold, 11-fold, and 11-fold, respectively, in the mutant (Fig. 4A). The *SFL1* gene, which has been suggested to play the opposite role to that of *SFL2* in hypha formation (13, 30), was upregulated 18-fold (Fig. 4A). The gene most dramatically upregulated in the *sfl2*Δ mutant was *OP4* (520-fold), the first gene to be identified as opaque specific (31). Three other genes implicated in switching, *SSN6* (32), *TUP1* (33), and *CDR3* (34), were also upregulated as well as the genes *CAG1*, *NRG1*, *BRG1*, *RFG1*, and *ZCF21* (Fig. 4A). *NDT80* was downregulated in opaque cells of the *sfl2*Δ mutant (Fig. 4A). Expression of *MTL***a***1* and *MTLα2*, which encode the **a**1-α2 corepressor of both switching and mating (4, 10, 11), were relatively unaffected by the white-to-opaque switch (Fig. 4A).

### Opaque stability of a/α *sfl2*Δ mutants.

**FIG 5:**
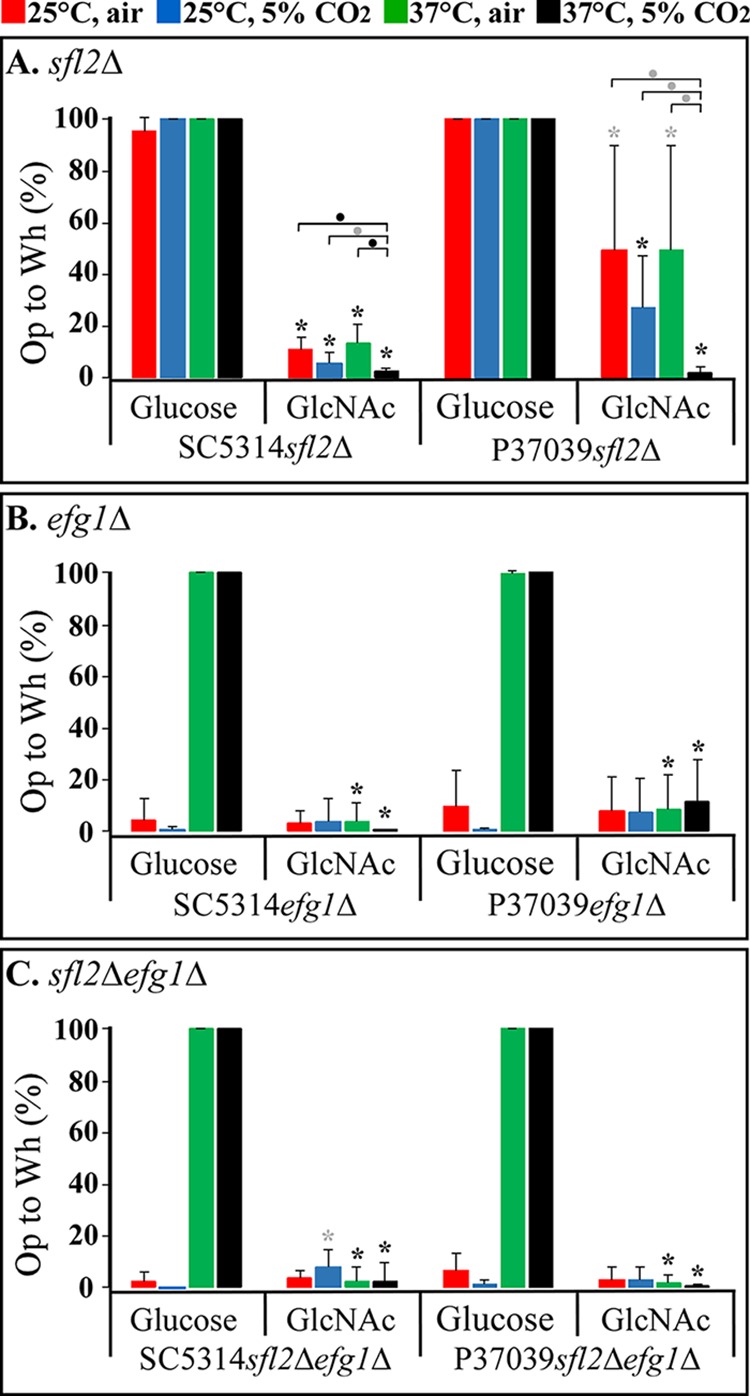
Opaque-to-white switching, a measure of opaque cell stability of the **a**/α SC5314 and **a**/α P37039 mutants under the eight sets of environmental conditions. (A to C) *sfl2Δ* mutants (A), *efg1Δ* mutants (B), and *sfl2Δ efg1Δ* mutants (C). Statistical significance was determined using Student’s *t* test analysis. Switching frequencies that are statistically significantly different between cells grown on glucose agar versus on GlcNAc agar under the four sets of conditions are indicated with asterisks, while switching frequencies that are significantly different for cells grown at 25°C in air, at 25°C in 5% CO_2_, and at 37°C in air versus at 37°C in 5% CO_2_ are indicated by solid circles. A gray asterisk or solid gray circle indicates 0.001 < *P* < 0.05. A black asterisk or solid black circle indicates 1 × 10^−35^ < *P <* 0.001.

The stability of the opaque phenotype (i.e., switching from opaque back to white) of the *sfl2*Δ mutants was then tested under the eight sets of conditions. When opaque cells of the mutants SC5314*sfl2*Δ and P37039*sfl2*Δ were plated on glucose-containing agar, there was mass conversion (95 to 100%) to white under all four sets of conditions (Fig. 5A and Table 3). On GlcNAc-containing agar under all four sets of conditions, switching from opaque to white of both *sfl2*Δ mutants occurred, but at reduced frequencies and with nuances. First, in both mutant strains, switching from opaque to white on GlcNAc agar was lowest at 37°C in 5% CO_2_ (Fig. 5A and Table 3), the same set of conditions that supported the highest frequency of white-to-opaque switching (Fig. 1A and Table 1). Second, under the remaining three sets of conditions on GlcNAc agar (25°C, air; 25°C, 5% CO_2_; 37°C, air), the frequencies of opaque to white switching were higher than at 37°C in 5% CO_2_ (Fig. 5A and Table 3). Together, these results indicate that GlcNAc not only supports switching but also stabilizes the opaque phenotype of **a**/α *sfl2*Δ cells and that 5% CO_2_ and high temperature (37°C), in combination, enhance GlcNAc stabilization. Glucose, on the other hand, induces mass conversion to white.

**TABLE 3:** Opaque-to-white switching, a measure of opaque phase stability, of **a**/α *sfl2*Δ, *efg1*Δ, and *sfl2*Δ *efg1*Δ mutants^*a*^

Strain	Carbon source	25°C, air		25°C, 5% CO_2_		37°C, air		37°C, 5% CO_2_	
		Total col. no.^*b*^	Frequency (%)^*c*^	Total col. no.	Frequency (%)	Total col. no.	Frequency (%)	Total col. no.	Frequency (%)
SC5314 *sfl2*Δ	Glucose	1,458	95.2 ± 5.6	1,407	100	1,511	100	1,504	100
	GlcNAc	1,522	11.2 ± 4.8	1,466	5.6 ± 4.0	1,560	13.2 ± 7.5	1,882	2.3 ± 1.3
SC5314 *efg1*Δ	Glucose	648	4.4 ± 8.1	700	0.9 ± 1.0	743	100	659	100
	GlcNAc	792	3.3 ± 4.3	774	3.9 ± 8.8	786	3.4 ± 7.7	964	0.2 ± 0.4
SC5314 *sfl2*Δ*efg1*Δ	Glucose	753	2.6 ± 3.3	741	0.0 ± 0.0	819	100	864	100
	GlcNAc	847	3.4 ± 3.1	922	7.7 ± 6.9	843	2.4 ± 5.2	1,092	2.6 ± 7.2

P37039 *sfl2*Δ	Glucose	660	100	677	100	703	100	679	100
	GlcNAc	737	49.6 ± 40.4	729	27.0 ± 20.2	681	49.3 ± 40.8	1,042	1.9 ± 2.1
P37039 *efg1*Δ	Glucose	696	9.5 ± 13.7	675	0.5 ± 0.8	519	99.5 ± 1.1	448	100
	GlcNAc	678	8.0 ± 13.3	693	7.5 ± 13.0	697	8.4 ± 13.4	944	11.4 ± 16.4
P37039 *sfl2*Δ*efg1*Δ	Glucose	632	6.4 ± 7.0	651	1.3 ± 2.0	724	100.0	743	100
	GlcNAc	727	2.7 ± 5.1	727	2.8 ± 5.1	724	1.6 ± 3.0	868	0.3 ± 0.8

a Switching was assessed under the eight sets of conditions, including all permutations of carbon source (glucose versus GlcNAc), temperature (25°C versus 37°C), and CO_2_ level (air [0.04% CO_2_] versus 5% CO_2_).

b Total colony number (Total col. no.) represents the total number of colonies pooled for the experiments.

c The frequencies of opaque to white switching are presented as the mean % ± standard deviations for ≥ 3 independent experiments.

### White-to-opaque switching by a/α *efg1*Δ mutants.

**FIG 6:**
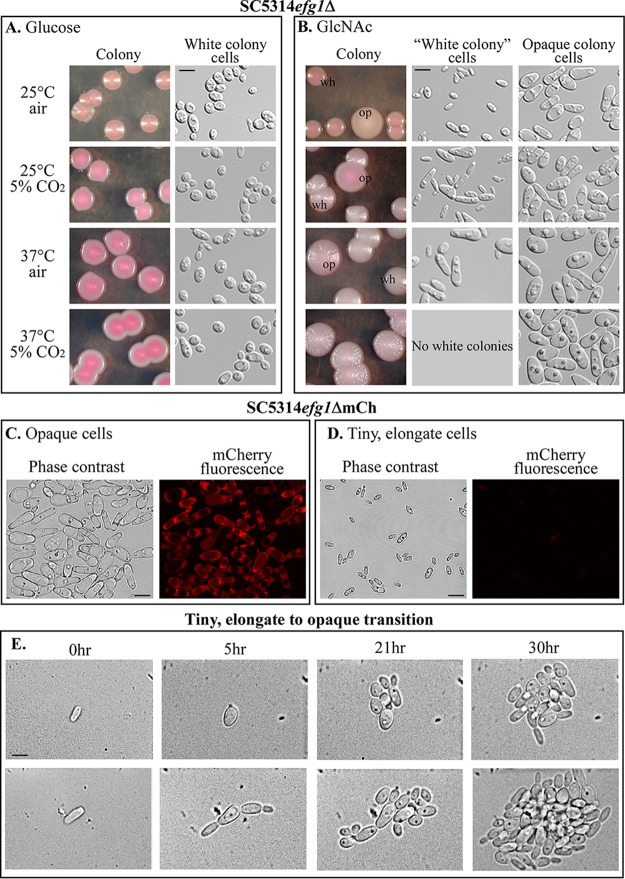
Colony and cell phenotypes of **a**/α SC5314*efg1Δ* mutants. (A) Representative images of colonies and cells of the SC5314*efg1Δ* strain on glucose agar under the four sets of environmental conditions. (B) Representative images of colonies and cells of the SC5314*efg1Δ* strain on GlcNAc agar under the four sets of environmental conditions. (C) Phase contrast and fluorescent images of cells in opaque colonies formed by the SC5314*efg1Δ*mCh strain, harboring the *mCherry* gene, under regulation of the opaque-specific *OP4* promoter. (D) Phase contrast and fluorescent images of tiny, elongated cells in white colonies formed by the SC5314*efg1*ΔmCh mutant. (E) Transition from the tiny, elongate cell phenotype to the opaque cell phenotype. Bars, 5 µm.

It has been shown that the transcription factor Sfl2 depends upon Efg1 for the activation of hypha formation (13, 30) and in coordination with Efg1 regulates the TCA cycle in response to high CO_2_ (35). Sfl2 has also been shown to bind to several of the same gene promoters (30). To assess the role of Efg1 in **a**/α switching, we generated the **a**/α *efg1*Δ mutants, SC5314*efg1*Δ and P37039*efg1*Δ (Table S1), and analyzed switching in the two mutants under the same eight sets of conditions. As was the case for both **a**/α *sfl2*Δ mutants, neither of the **a**/α *efg1*Δ mutants underwent white-to-opaque switching on glucose agar under the four permutations of temperature and CO_2_ (Table 1 and Fig. 1B). Cells in *efg1*Δ white colonies formed on glucose agar under the four sets of conditions and exhibited the standard round to ovoid white yeast phase phenotype (20, 21). However, on GlcNAc agar, there were fundamental differences in cell phenotype between the **a**/α *efg1*Δ and **a**/α *sfl2*Δ mutants. On GlcNAc agar at 25°C in air or 5% CO_2_, white cells of both **a**/α *efg1*Δ mutants underwent low to negligible levels of switching to opaque (0 to 5%), as assessed by colony phenotype (Table 1 and Fig. 1B). The few opaque colonies that formed contained cells exhibiting the signature opaque cell phenotype (Fig. 5B). These opaque cells stained selectively for pimples, with the antipimple polyclonal antibody (Fig. 3E and F). However, on GlcNAc agar at 25°C in air, morphologically white colonies contained a majority of a tiny, elongate phenotype and a minority of yeast phase cells, and on GlcNAc agar at 25°C in 5% CO_2_, white colonies contained mixtures of tiny, elongate cells and opaque cells (Fig. 6B). On GlcNAc agar at 37°C in air, **a**/α *efg1*Δ cells in white colonies contained a minority of yeast phase cells and a majority of opaque cells (Fig. 6B). On GlcNAc agar at 37°C in 5% CO_2_, the conditions that supported mass conversion to opaque in **a**/α *sfl2*Δ mutants (Fig. 1A and Table 1), cells of the two **a**/α *efg1*Δ mutants switched en masse (100%) to opaque (Table 1 and Fig. 1B) and exhibited the signature opaque cell phenotype (Fig. 6B). Opaque cells of **a**/α *efg1*Δ mutant strains, in which mCherry was placed under the control of the opaque-specific *OP4* promoter, were highly fluorescent (Fig. 6C).

### Tiny, elongate cell phenotype.

We tested whether tiny, elongate cells of strains SC5314*efg1*ΔmCh and P37039*efg1*ΔmCh, in which *mCherry* was under the regulation of the promoter of the opaque-specific gene *OP4* (31) (Table S1), were fluorescent. While opaque cells of these strains fluoresced red (Fig. 6C), the tiny, elongate cells exhibited negligible fluorescence (Fig. 6D). Tiny, elongate cells were also tested for staining by the polyclonal antipimple antibody (22). The antibody did not stain tiny, elongate cells (Fig. 3I and J, respectively). Because tiny, elongate cells were mixed with opaque cells in *efg1*Δ cultures grown under conditions suboptimal for mass conversion to opaque, we tested whether the tiny, elongate cells, when transferred to GlcNAc medium at 37°C in 5% CO_2_, formed opaque cells. More than 10 tiny, elongate cells dispersed under agarose overlays were monitored microscopically for 30 h. The individual tiny, elongate cells grew into vacuolated opaque cells, which then continued to multiply in the opaque phase to form opaque microcolonies (Fig. 6E).

### Gene expression in *efg1*Δ opaque cells.

The same set of genes analyzed in **a**/α SC5314*sfl2*Δ opaque cells by qRT-PCR were analyzed in **a**/α SC5314*efg1*Δ opaque cells formed on GlcNAc agar at 37°C in 5% CO_2_ (Fig. 4B). Opaque cells of both mutants exhibited dramatic increases in *WOR1* expression (322- and 135-fold, respectively) and *OP4* expression (525- and 1,197-fold, respectively) (Fig. 4A and B, respectively). Opaque cells of both *sfl2*Δ and *efg1*Δ mutants also exhibited increases in *WOR2*, *WOR3*, *WOR4*, *SFL1*, *SSN6*, *CDR3*, and *ZCF21* (Fig. 4A and B). Opaque cells of the *efg1*Δ mutant did not exhibit the increases in the expression of *AHR1*, *TUP1*, *CAG1*, *NRG1*, *BRG1,* and *RFG1* observed in the *sfl2*Δ mutant (Fig. 4A). On the other hand, expression of *NDT80* increased, and expression of *CZF1* and *UME6* decreased in opaque cells of the *efg1*Δ mutant (Fig. 4B), unlike expression in the *sfl2*Δ mutant (Fig. 4A).

### Opaque stability of the *efg1*Δ mutants.

The *efg1*Δ mutants were tested for opaque cell stability (i.e., opaque-to-white switching) under the eight sets of conditions. On glucose agar at 25°C in air or 5% CO_2_, opaque cells of both *efg1*Δ mutants were relatively stable (Fig. 5B and Table 3). This differed markedly from opaque cells of the two *sfl2*Δ mutants, which switched en masse (95 to 100%) (Fig. 5A and Table 3). On glucose agar at 37°C in air or 5% CO_2_, opaque cells of both *efg1Δ* mutants underwent mass conversion to white (i.e., were completely unstable) (Fig. 5B and Table 3), results similar to those for opaque cells of the *sfl2*Δ mutants (Fig. 5A and Table 3). On GlcNAc agar, the *efg1Δ* mutants were moderately stable under all four sets of conditions, switching from opaque to white at frequencies between 0.2 and 11% (Fig. 5B and Table 3). For both strains, opaque stability under the four conditions on GlcNAc agar, was on average greater for the *efg1Δ* mutants than for the *sfl2Δ* mutants.

### White-to-opaque switching by a/α *sfl2*Δ *efg1*Δ mutants.

To assess whether the effects of the mutations of *SFL2* and *EFG1* are synergistic or whether either is dominant under one or more of the eight sets of conditions, the double **a**/α mutants SC5314*sfl2Δefg1Δ* and P37039*sfl2Δefg1Δ* (Table S1) were tested under the eight sets of conditions. The absence of switching on glucose agar under all four permutations of temperature and atmosphere was the same as that for the individual *sfl2*Δ and *efg1*Δ mutants (Table 1 and Fig. 1A to C). The patterns of switching under the four sets of conditions on GlcNAc agar, including the formation of tiny, elongate cells, was similar to that of the individual *efg1*Δ mutants (Table 1 and Fig. 1C). Mass conversion to opaque on GlcNAc agar at 37°C in 5% CO_2_ was similar to that of both of the single mutants (Fig. 1C). These results indicate that in the double mutant, the *efg1*Δ mutation overrides the *sfl2*Δ mutation.

### Opaque stability of the a/α *sfl2*Δ *efg1*Δ mutants.

The stability of opaque cells of the **a**/α *sfl2*Δ *efg1*Δ mutants on both glucose and GlcNAc agar under the four sets of environmental conditions mimicked that of the *efg1*Δ mutants, rather than that of the *sfl2*Δ mutants (Fig. 5 and Table 3). These results indicate that just as in the case of the white-to-opaque transition, the *efg1*Δ mutation overrides the *sfl2*Δ mutation in the opaque-to-white transition of the *sfl2*Δ *efg1*Δ mutants.

### Complementation of the *sfl2*Δ and *efg1*Δ mutants.

To test whether the switching phenotypes on GlcNAc agar exhibited by the two *sfl2*Δ mutants and the two *efg1*Δ mutants were due to the targeted mutated genes, all were complemented with the wild-type gene. Complementation was targeted to the native locus. Switching data of the complemented strains are provided in Table 4 for GlcNAc agar, 37°C, 5% CO_2_, and for all eight sets of conditions in Table S5. The addition of one copy of *SFL2* to each of the two *sfl2*Δ mutants (SC5314*sfl2*Δ and P37039*sfl2*Δ) resulted in a reduction, but not in the elimination, of switching, the latter representing the parental wild-type phenotype (Table 4 and Table S5). The reduction observed in the *sfl2*Δ mutants receiving one copy of *SFL2* was similar to that of the heterozygous deletion derivative (Table 4 and Table S5). However, additions of two copies of *SFL2* to the native allelic loci resulted in the elimination of switching, presumably a gene dosage effect (Table 4 and Table S5). In contrast, addition of one *EFG1* copy to both *efg1*Δ mutants resulted in the complete elimination of white-to-opaque switching on GlcNAc agar at 25°C or 37°C in 5% CO_2_ (Table 4 and Table S5). These results demonstrate that the switching phenotypes of the two *sfl2*Δ mutants and the two *efg1*Δ mutants analyzed in detail were the result of the deletion of the targeted genes.

**TABLE 4:** Complementation of the *SFL2* and *EFG1* in the *sfl2Δ* and *efg1Δ* mutants reestablishes wild-type switching phenotype^*a*^

Strain	Complementation	Total no. of colonies	Frequency of white-to-opaque switching (%)
SC5314 *SFL2/SFL2*		1,401	0
SC5314 *sfl2*Δ*/sfl2*Δ		2,544	93.8 ± 5.8
SC5314 *SFL2/sfl2*Δ		1,997	5.3 ± 1.2
SC5314 *sfl2*Δ*/sfl2*Δ	*SFL2*	2,471	11.7 ± 2.7
SC5314 *sfl2*Δ*/sfl2*Δ	*SFL2*/*SFL2*	2,471	0

P37039 *SFL2/SFL2*		1,370	0
P37039 *sfl2*Δ*/sfl2*Δ		2,243	99.6 ± 0.6
P37039 *SFL2/sfl2Δ*		1,728	9.3 ± 4.9
P37039 *sfl2*Δ*/sfl2*Δ	*SFL2*	2,002	8.7 ± 2.7
P37039 *sfl2*Δ*/sfl2*Δ	*SFL2*/*SFL2*	1,707	0

SC5314 *EFG1/EFG1*		1,401	0
SC5314 *efg1*Δ*/efg1*Δ		2,341	100
SC5314 *efg1*Δ*/efg1*Δ	*EFG1*	1,107	0

P37039 *EFG1/EFG1*		1,370	0
P37039 *efg1*Δ*/efg1*Δ		1,849	95.8 ± 7.2
P37039 *efg1*Δ*/efg1*Δ	*EFG1*	970	0

a The frequency of switching was assessed on GlcNAc agar at 37°C in 5% CO_2_. For complete developmental phenotypes under the eight sets of environmental conditions, see Table S5 in the supplemental material. It should be noted that one copy of *EFG1* was required to complement the *efg1Δ/efg1Δ* mutants, and two copies of *SFL2* were required to complement the *sfl2Δ/sfl2Δ* mutants.

### Inhibition versus induction by the sugar sources.

**FIG 7:**
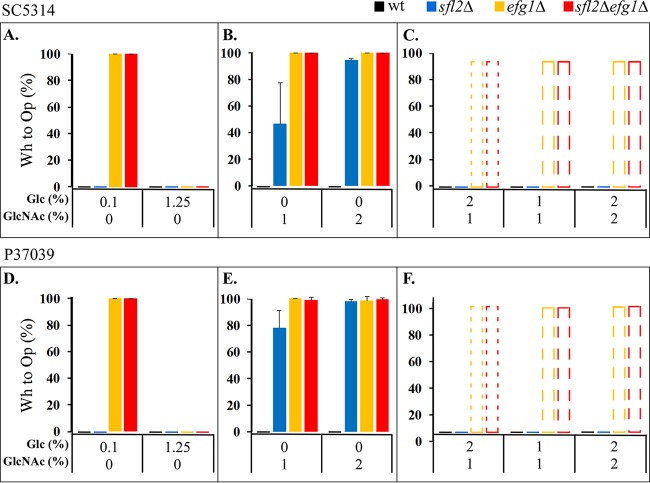
Glucose is an inhibitor and GlcNAc is an inducer of white-to-opaque switching. Switching of the parental **a**/α wild-type SC5314 and P37039 and their *sfl2Δ*, *efg1Δ*, and *sfl2Δ efg1Δ* mutants was tested at 37°C in 5% CO_2_ on sLee’s agar with the indicated combinations of glucose and GlcNAc. (A and D) Two glucose concentrations. (B and E) Two GlcNAc concentrations. (C and F) Combinations of glucose and GlcNAc. The bars indicate mixtures of opaque (majority) and white (minority) cells. The error bars in panels A, B, D, and E represent standard derivatives from three experiments. Glc, glucose; wt, wild type.

The preceding results led us to the hypothesis that glucose may be a repressor and GlcNAc an inducer of white-to-opaque switching in the mutants. We tested whether glucose was a repressor by decreasing the concentration from 1.2%, the standard concentration in supplemented Lee’s medium (19, 36), to 0.1%, close to the average concentration in the gut (37), and then analyzing switching from white to opaque at 37°C in 5% CO_2_. If glucose acted as a repressor, the decrease in glucose concentration would result in an increase in the frequency of white-to-opaque switching. Neither the parent **a**/α strains nor the two derivative *sfl2*Δ mutants underwent switching on 0.1% glucose agar at 37°C in 5% CO_2_ (Fig. 7A and D). However, both of the *efg1*Δ mutants and both of the *sfl2*Δ *efg1*Δ mutants, which did not switch in 1.25% glucose (Fig. 1 and Table 1), underwent mass conversion from white to opaque (100%) on 0.1% glucose agar (Fig. 7A and D). The opaque colonies formed by the **a**/α *efg1*Δ and *efg1*Δ *sfl2*Δ mutants on 0.1% glucose agar contained cells with signature opaque morphologies. Therefore, mass conversion from white to opaque by the **a**/α *efg1*Δ and *sfl2*Δ *efg1*Δ mutants was due in this case to the reduction of glucose concentration, suggesting that the standard concentration of glucose (1.25%) used *in vitro* represses switching of the **a**/α *efg1*Δ mutants, but not the **a**/α *sfl2*Δ mutants.

For our previous analyses on GlcNAc agar, a concentration of 2% was routinely employed. We therefore first tested whether reducing the concentration of GlcNAc by half affected switching. As previously demonstrated here (Fig. 1 and Table 1), a GlcNAc concentration of 2% at 37°C in 5% CO_2_ did not support white-to-opaque switching of the two parental **a**/α strains but supported mass conversion (95% to 100%) from white to opaque of all three mutants (*sfl2*Δ, *efg1*Δ, and *sfl2*Δ *efg1*Δ mutants) (Fig. 7B and E). A 1% concentration of GlcNAc, like 2%, also resulted in mass conversion of the *efg1*Δ and *sfl2*Δ *efg1*Δ mutants (Fig. 7B and E). However, for both *sfl2*Δ mutants, 1% GlcNAc resulted in a reduced frequency of switching (48% and 78%, respectively) (Fig. 7B and E, respectively). These results did not exclude the possibility that the *efg1*Δ and *sfl2*Δ *efg1*Δ mutants underwent mass conversion as a default response in the absence of glucose inhibition. However, the decrease in switching by both *sfl2*Δ mutants in 1% GlcNAc supported an inductive role.

If glucose is an inhibitor and GlcNAc is an inducer of switching in **a**/α strains that carry an *sfl2*Δ or *efg1*Δ mutation, then when the two sugars are present in combination, the effect of one may be dominant over that of the other. We therefore tested the following combinations of glucose and GlcNAc at 37°C in 5% CO_2_: 2%:0%, 1%:1%, 1%:0%, and 2%:2%. For all combinations, white-to-opaque switching did not occur in the wild-type **a**/α strains or their *sfl2*Δ derivatives. The results indicated that for *sfl2*Δ mutants, glucose repression overrode GlcNAc induction. However, for the *efg1*Δ derivatives, the results were more complex. At glucose-to-GlcNAc ratios of 2%:1%, the morphologically “white” *efg1*Δ and *sfl2*Δ *efg1*Δ colonies contained a majority of yeast phase cells, but a minority of opaque phase cells as well (dotted bars in Fig. 7C and F; Fig. S1). At ratios of 1%:1% and 2%:2%, morphologically “white” colonies contained a majority of opaque cells and a minority of white cells (dashed bars in Fig. 7C and F; Fig. S1). None of the morphologically “white” colonies formed by the *efg1*Δ mutant on the three sugar mixtures contained tiny, elongate cells, since the formation of this phenotype is repressed by high temperature (37°C). These results indicate that when glucose is the sole carbon source, it acts as a repressor of white-to-opaque switching by *sfl2*Δ and *efg1*Δ mutant cells, when GlcNAc is the sole carbon source, it functions as an inducer, and when in combination, GlcNAc induction of white to opaque partially overrides glucose repression of switching.

10.1128/mSphere.00703-18.1 FIG S1 White-to-opaque switching of the SC5314*efg1*Δ mutant on various combinations of glucose and GlcNAc at 37°C in 5% CO_2_. Cells were from the morphologically white colonies grown on agar for 3 days. Each of the combinations of carbon source are indicated at the top of each pair of images. Scale bar, 5 μm. Glc, glucose; Nac, *N*-acetylglucosamine. Download FIG S1, TIF file, 2.9 MB. Copyright © 2019 Park et al. 2019 Park et al. This content is distributed under the terms of the Creative Commons Attribution 4.0 International license.

## DISCUSSION

To assess by mutational analyses the roles of Sfl2 and Efg1 in repressing white-opaque switching in **a**/α strains of Candida albicans under different sets of environmental conditions, two parental **a**/α strains were selected that did not undergo switching under any of the eight sets of environmental conditions, which included all permutations of carbon source, temperature, and CO_2_ level. Xie et al. (7) found that approximately a third of natural **a**/α strains switched from white to opaque on GlcNAc agar at 25°C in 5% CO_2_. Here, we have shown that with 1.25% glucose as the carbon source, neither the wild type nor the three tested mutants of both strains, the *sfl2*Δ, *efg1*Δ, and *sfl2*Δ *efg1Δ* mutants, switched under any of the four permutations of temperature and CO_2_. When the concentration of glucose was lowered to 0.1%, growth slowed and the two *efg1*Δ mutants (*efg1*Δ and *sfl2*Δ *efg1Δ*) underwent mass conversion (>95%) to opaque, suggesting that high levels of glucose repressed white-to-opaque switching in both mutants. We also found that when cells of the *efg1*Δ mutants were grown on mixtures of glucose and GlcNAc, tiny, elongate cells did not form. Moreover, high GlcNAc (2%) appeared to induce white-to-opaque switching in the presence of high glucose (2%). These results suggest that high concentrations of glucose repress switching from white to opaque in **a**/α *sfl2*Δ and *efg1*Δ mutants and repress formation of tiny, elongate cells in *efg1*Δ mutants. Furthermore, the repression by glucose of white-to-opaque switching appears to be reversed by the addition of GlcNAc. It should be noted that Doedt and coworkers (38) have demonstrated that Efg1 affects expression of genes involved in sugar metabolism, acting as an inducer of genes involved in glycolysis, the citric acid cycle, and cell wall biosynthesis. On the other hand, Znaidi and coworkers (30) using ChIP binding assays did not identify a similar general increase in the expression of genes directly involved in sugar metabolism or the citric acid cycle in an *sfl2*Δ mutant but did find that many of the binding sites of *Sfl2* were also binding sites of *Efg1*. These results do not distinguish between receptor-mediated mechanisms versus catabolic mechanisms for either glucose repression or GlcNAc induction.

The stability of opaque cells formed by the *sfl2*Δ and *efg1*Δ mutants (*efg1*Δ and *sfl2*Δ *efg1Δ* mutants) differed on glucose agar at 25°C. At 37°C, both the *sfl2*Δ and *efg1*Δ mutants underwent mass conversion from opaque to white in air or 5% CO_2_, but at 25°C, *sfl2*Δ mutants underwent mass conversion, but *efg1*Δ mutants were highly stable. This was true for the mutants derived from strain SC5314 and from strain P37039. These results demonstrate a fundamental difference in the stability of the opaque phenotype between *sfl2*Δ and *efg1*Δ mutants at physiological temperature (37°C), suggesting that *efg1*Δ mutants, but not *sfl2*Δ mutants, might stably express the opaque phenotype in the host.

The concentration of free glucose in the intestine varies dramatically in mammals, averaging 0.2% under normal feeding conditions (37). This concentration is well below that which has been used in the medium to grow C. albicans *in vitro*. GlcNAc is also found in the intestine, a product of mucin digestion by bacterial enzymes (39–41). Accurate measures of free GlcNAc could not be found. On GlcNAc agar, mass conversion from white to opaque occurred in both *sfl2*Δ and *efg1*Δ mutants at 37°C in 5% CO_2_. However, under the other three sets of conditions (25°C in air or CO_2_; 37°C in air), there were major differences in cellular phenotype. At 25°C in air, no switching to opaque occurred in any of the three mutants, but while white *sfl2*Δ cells continued to form white yeast phase cells, the *efg1*Δ and *sfl2*Δ *efg1*Δ mutants formed tiny, elongate cells. On GlcNAc agar at 25°C in 5% CO_2_, the *efg1*Δ mutants formed mixtures of tiny elongate and opaque phase cells. At 37°C in 5% CO_2_, however, the majority of white cells of all three mutants (*sfl2*Δ, *efg1*Δ, and *sfl2*Δ *efg1Δ* mutants) uniformly formed opaque cells. We show here by single cell analysis that if tiny, elongate cells formed at 25°C are placed under opaque-inducing conditions at 37°C, they differentiate into opaque cells. Similar small, elongate cells have been reported in two previous studies. Langford et al. (42) found that when *EFG1* and *CZF1* were both deleted in an **a**/α strain originally derived from SC5314 **a**/α, they formed a tiny, elongate phenotype at 30°C, but not 37°C. Tao et al. (43) reported that the **a**/α strain BJ1097 switched between three colony phenotypes on YPD agar, white, opaque, and gray, and that a majority of cells in the gray colonies formed small, elongate cells that lacked cell wall pimples. It seems likely that these small, elongate cells represent the same phenotype we have found expressed by **a**/α *efg1*Δ mutants.

Our results indicate that employing one set of environmental conditions may be inadequate for assessing the roles of many TFs in the process of white-opaque switching. To this point, a recent review by Noble et al. (44) on “switching and functional plasticity in the human host” emphasizes the importance of environmental conditions. Although we have focused on two transcription factors, it seems likely that mutational analyses of a number of additional transcription factors, including those regulated by Sfl2 and Efg1, will reveal similar as well as dissimilar patterns of switching, under different patterns of environmental conditions. In light of the results of Xie et al. (7) demonstrating that more than a third of tested natural **a**/α strains switch to opaque under inducing conditions, it seems likely that mutations in genes involved in the modulation of switching may have accumulated among natural strains of C. albicans, which has a highly clonal population structure. These different mutants may indeed provide specialized adaptive advantages in commensalism and pathogenesis. Indeed, switching of *MTL*-homozygous strains to the opaque phenotype has been shown to affect the capacity to colonize skin (45). It is likely no coincidence that the *in vitro* conditions necessary for inducing mass conversion from white to opaque in the two **a**/α mutants examined, the *sfl2*Δ mutant and *efg1*Δ mutant, correspond to the most common physiological conditions C. albicans experienced in the human host.

## MATERIALS AND METHODS

### Strains and media.

The C. albicans strains used in this study are described in Table S1 in the supplemental material. The strains were maintained at room temperature on nutrient agar containing YPD (1% yeast extract, 2% peptone, 2% glucose) medium or agar containing supplemented Lee’s (sLee's) medium, which contains 1.25% glucose (36). The latter (sLee's agar) also contained 5 μg/liter of phloxine B, which differentially stains opaque colonies light red (21). Opaque cells of the *sfl2*Δ, *efg1*Δ, and *sfl2*Δ *efg1*Δ mutant strains were maintained on nutrient agar containing sLee’s-GlcNAc (2%) medium (GlcNAc agar) with phloxine B due to their instability in the presence of glucose. Prior to use, white cells were spread on fresh YPD or sLee’s agar and grown for 2 days at 30°C or 5 days at 25°C in air. Escherichia coli strain XL1-Blue (Agilent Technologies, TX, USA), used for the generation and maintenance of plasmids, was grown in LB medium (1% tryptone, 0.5% yeast extract, 1% NaCl) plus 100 µg/ml ampicillin.

### Construction of plasmids and generation of C. albicans strains.

To delete the *EFG1*, *SFL2*, and *WOR1* genes, in the wild-type C. albicans **a**/α strains SC5314 and P37039 (Table S1), we first generated the plasmids pEFG1SM, pSFL2SM, and pWOR1SM, respectively. To generate the plasmids, the 5′ and 3′ regions of the genes were amplified by PCR with the primer pairs listed in Table S2 using genomic DNA from strain SC5314 as the template. The 5′ and 3′ PCR-amplified DNA fragments of the genes and plasmid pSFS2, which contains a C. albicans-adapted gene deletion cassette (46), were digested with the appropriate restriction enzymes (the target sequences of the enzymes are underlined in the sequences in Table S2). The DNA fragments into the vector plasmid were then ligated to clone the plasmids that have the gene deletion cassette flanked by the 5′ and 3′ regions of the genes. To reintroduce a wild-type *EFG1* into its native locus in the *efg1*Δ mutants for complementation, we generated the pEFG1C plasmid. A coding region of the *EFG1* gene was first amplified by PCR with the primers EFG1-F/-R (Table S2) and placed under the Tet-On promoter in the pNIM1 plasmid (47), resulting in the pNIM-EFG1 plasmid. A PstI/SalI-digested DNA fragment of the *EFG1* and *CaSAT1* selection marker genes from the pNIM-EFG1plasmid was fused by ligation with a 5′ promoter region from the pEFG1SM plasmid and a 3′ region of the *EFG1* gene, which were amplified by PCR with the primer pair EFG1-3´F1/-3´R (Table S2), into the pBluescript vector plasmid of pSFS2 (46). This resulted in pEFG1C. To reintroduce a wild-type copy of *SFL2* into the native locus in the *sfl2*Δ mutants for complementation, we similarly generated the pSFL2SC plasmid. A coding region of the *SFL2* gene was first amplified by PCR with the primers SFL2-F/-R (Table S2) and placed under the Tet-On promoter in the pNIM1 plasmid (47), resulting in the pNIM-SFL2 plasmid. A PstI/SalI-digested DNA fragment of the *SFL2* and *CaSAT1* genes from the pNIM-SFL2 plasmid was fused by ligation with a 5′ promoter region and a 3′ region of the *SFL2* gene, which were amplified by PCR with the primer pairs, SFL2-3´F/-R1 and SFL2-3´F1/-3´R (Table S2), respectively, into the vector plasmid of pSFS2 (46). To complement both copies of *SFL2*, plasmid pSFL2HC was generated by swapping the *CaSAT1* gene in plasmid pSFL2SC with the *CaHygB* gene. To create plasmid pmCherry-HygB, the *mCherry* gene, which was amplified by PCR with primers mCherry-F/-R from plasmid pmCherry-SAT (48), was digested with SalI and BamHI, and inserted into the SalI/BglII-digested vector pGFP-HygB (49). All sequences of the amplified DNA fragments were verified by sequencing after cloning into the plasmids. Plasmids pGFP-SAT, pmCherry-SAT (48), and pGFP-HygB (49) were used to generate strains for mating experiments. Deletion of genes was performed as described by Reuss et al. (46). The gene deletion, complementation, and insertion cassettes from the generated plasmids were used to transform the two C. albicans **a**/α strains SC5314 and P37039 (Table S1). For selection of cells transformed with the gene deletion or insertion cassettes, YPD agar containing 200 µg/ml of nourseothricin or 1 mg/ml hygromycin B was employed, depending on the selection marker. *CaSAT1* and *CaHygB* conferred resistance to nourseothricin and hygromycin B, respectively. Correct integration of the gene deletion and insertion cassettes, and deletion of the genes, were verified by PCR.

10.1128/mSphere.00703-18.3 TABLE S2 Primers used in this study. Download Table S2, DOCX file, 0.02 MB. Copyright © 2019 Park et al. 2019 Park et al. This content is distributed under the terms of the Creative Commons Attribution 4.0 International license.

10.1128/mSphere.00703-18.4 TABLE S3 The **a**/α *sfl2*Δ *wor1*Δ double knockout mutants did not switch to opaque cells. The total colony numbers were pooled from at least three independently performed experiments. Download Table S3, DOCX file, 0.02 MB. Copyright © 2019 Park et al. 2019 Park et al. This content is distributed under the terms of the Creative Commons Attribution 4.0 International license.

10.1128/mSphere.00703-18.5 TABLE S4 White-to-opaque switching in different concentrations of glucose and GlcNAc, as well as in combinations of the two sugars. The agar cultures were incubated for 3 days at 37°C in 5% CO_2_, prior to analysis of colony and cell morphology. Opaque-sectored colonies were counted as opaque. The data represent the means ± standard deviations from two or more experiments. Download Table S4, DOCX file, 0.01 MB. Copyright © 2019 Park et al. 2019 Park et al. This content is distributed under the terms of the Creative Commons Attribution 4.0 International license.

10.1128/mSphere.00703-18.6 TABLE S5 White-to-opaque switching frequency of the *SFL2* and *EFG1* complemented strains. The total colony numbers of at least two independently performed experiments were pooled. Download Table S5, DOCX file, 0.02 MB. Copyright © 2019 Park et al. 2019 Park et al. This content is distributed under the terms of the Creative Commons Attribution 4.0 International license.

### Switching assays.

White-opaque switching was assessed on glucose agar or GlcNAc agar under four different sets of environmental conditions: 25°C, air; 25°C, 5% CO_2_; 37°C, air; and 37°C, 5% CO_2_. To assess switching from white to opaque, yeast (white) cells from YPD suspension cultures grown overnight at 25°C were plated at approximately 200 cells per 10-cm petri dish on glucose or GlcNAc agar. At 25°C in air or 5% CO_2_, agar cultures were incubated 5 days. At 37°C in air or 5% CO_2_, agar cultures were incubated for 3 days. For white-to-opaque switching, opaque-sectored as well as homogeneous opaque colonies were both counted as opaque. At the colony level, colonies formed by *efg1*Δ and *sfl2*Δ *efg1*Δ mutants at 25°C in 5% CO_2_ and at 37°C in air were scored as “white” but contained mixtures of tiny, elongate cells and opaque cells or mixtures of white and opaque cells, respectively. These observations are noted in Results. Therefore, random colonies, both white and opaque, and random opaque sectors were examined microscopically for cellular phenotypes in every experiment. To assess switching from opaque to white under the eight sets of environmental conditions, cells from homogeneous opaque colonies that formed on GlcNAc agar at 25°C in air for 5 days were plated on glucose or GlcNAc agar plates and analyzed for colony phenotype under the four sets of environmental conditions as described above for white-to-opaque switching. For opaque to white switching, white-sectored as well as homogeneous white colonies were scored as “white.”

### Quantitative RT-PCR.

To obtain RNA for performing quantitative reverse transcription-PCR (qRT-PCR), cells were harvested from colonies grown for 3 days on sLee's-GlcNAc at 37°C in 5% CO_2_ and then washed twice with water. Cells were then resuspended in RNAlater solution (Ambion, Life Technologies, Carlsbad, CA, USA) and incubated for 1 h at 4°C and stored at −80°C until use. RNA was extracted using the RNeasy minikit (Qiagen) according to the manufacturer’s instructions. The TURBO DNA-free kit (Ambion, Life Technologies, Carlsbad, CA, USA) was used to remove DNA contamination. Finally, RNA quality was confirmed to be higher than 9.5 RQI (RNA quality indicator) with the Experion RNA StdSens and HighSens Analysis kit (Bio-Rad). For qRT-PCR assays, cDNA was first generated from the RNA samples using the iScript cDNA synthesis kit (Bio-Rad) as recommended by the manufacturer. qRT-PCR assays were performed with a LightCycler 480 SYBR Green I Master mix (Roche), according to the manufacturer’s instructions. The transcripts were quantified using a Roche LightCycler480 real-time PCR detection system with SYBR green. The relative expression level of each gene was normalized to that of C. albicans *TDH3* (50, 51). Primer pairs used for qRT-PCR are listed in Table S2.

### Mating assay.

To assess the capacity of opaque cells of the **a**/α *sfl2*Δ mutants to mate, the hygromycin B-resistant *sfl2*Δ derivatives, SC5314*sfl2Δ*-Hr and P37039*sfl2*Δ-Hr, and the **a/a** and α/α nourseothricin-resistant derivatives, P37005-Sr (**a/a**) and WO-1-Sr (α/α) derived from the **a/a** strain P37005 and α/α strain WO-1 (Table S1), respectively, were employed. To measure control levels of mating between **a/a** and α/α opaque cells, opaque cells of the hygromycin B-resistant strain **a/a** P37005-Hr and the nourseothricin-resistant strain α/α WO-1-Sr (Table S1) were crossed. Opaque cell suspensions of the strains were obtained from 5 to 10 homogeneous opaque colonies that were grown on sLee's-GlcNAc agar medium for 5 days at 25°C in air. The numbers of cells in the opaque cell suspensions were counted, and 1 × 10^6^ opaque cells of each of the mating partners was mixed in 10 µl of water. To facilitate mating, 10-µl portions of the mating mixtures were spotted onto a nitrocellulose membrane placed on GlcNAc agar and incubated for 2 and 4 days at 25°C in air. The mating patches on the membrane were then resuspended in 1 ml of water, and 100-µl aliquots of the undiluted (10°) and diluted (10^−1^ and 10^−2^) cell suspensions plated on YPD selection agar containing 200 µg/ml of nourseothricin and 1 mg/ml of hygromycin B. To measure total CFU in the recovered mating suspensions, 100-µl aliquots of 10^−4^ and 10^−5^ dilutions were plated on YPD agar in the absence of selection molecules. Mating events and total CFU were scored after 3 days of incubation at 30°C, and mating frequencies were computed.

### Immunolocalization of the opaque-specific pimple marker.

Rabbit-derived polyclonal antipimple antiserum (22) was used to visualize the formation of opaque-specific pimples. Opaque cells were heat killed in a 65°C water bath for 1 h. Pelleted cells were then resuspended in phosphate buffer solution (PBS) supplemented with 10% normal goat serum to block nonspecific binding. A 1:50 dilution of rabbit serum was preabsorbed five times with heat-killed *MTL*-homozygous white cells to remove antibodies to surface antigens common to white and opaque cells (22). Following staining with the primary antiserum, cells were washed with PBS and treated with Alexa Fluor 488-tagged goat anti-rabbit secondary antibody (Jackson ImmunoResearch, West Grove, PA). Fluorescent images were captured using a Bio-Rad 2100 multiphoton LSCM. Differential interference contrast (DIC) images were taken using a Canon Rebel T3i digital camera.

### Imaging colonies and cells.

Colonies of strains, which were grown on agar plates under the indicated conditions in Results, were imaged through a stereo microscope equipped with a Nikon E990 digital camera. Cells from the colonies were imaged with a Canon Rebel T3i digital camera attached to a Bio-Rad Radiance 2000MP upright microscope with a 60× plan water immersion objective. Cells expressing *mCherry* were imaged with a Leica TCS SP8 confocal microscope and image processed with Image J software.

### Imaging the transition from the tiny, elongate phenotype to the opaque phenotype.

To follow the transition from individual tiny, elongate cells to opaque cells, at the cellular level, tiny, elongate cells of **a**/α *efg1*Δ were plated under a thin layer of agarose in GlcNAc medium in a glass-bottomed, gridded 30 µ-dish (ibidi GmbH, Germany) and then incubated for 30 h at 37°C in 5% CO_2_. At time points, cells were imaged with a Canon Rebel T3i digital camera attached to a Bio-Rad Radiance 2000MP upright microscope with a 60× plan water immersion objective. At least 10 tiny, elongated cells were individually followed until the cells developed microcolonies.
