# Supplementary material for: Roles of the Transcription Factors Sfl2 and Efg1 in White-Opaque Switching in a/α Strains of Candida albicans
Source: mSphere. 2019 Apr 17;4(2):e00703-18. doi: 10.1128/mSphere.00703-18 (PMC6470211; doi:10.1128/mSphere.00703-18)
Supplement: TABLE S1 [file mSphere.00703-18-st001.docx]

**TABLE S1** Strains used in this study. The two independently generated strains from the wild type **a**/α strains SC5314 and P37039 were indicated as A and B after the name of strains.

| **Strain** | **Parental strain** | **Genotype** | **Reference** |
| --- | --- | --- | --- |
|  |  |  |  |
| SC5314 | - | *MTL***a**/α, wild type | (1) |
| P37039 | - | *MTL***a**/α, wild type | (2) |
| P37005-Hr | 37005 | *MTL***a/a**, *OP4/op4::OP4p-GFP-CaHygB* | (3) |
| P37005-Sr | 37005 | *MTL***a/a**, *OP4/op4::OP4p-GFP-CaSAT1* | (4) |
| WO-1-Sr | WO-1 | *MTL*α/α, *OP4/op4::OP4p-GFP-CaSAT1* | This study |
| SC5314*SFL2*/*sfl2*Δ A, B | SC5314 | *MTL***a**/α, *SFL2*/*sfl2*::*FRT* | This study |
| SC5314*sfl2*Δ A, B | SC5314*SFL2*/*sfl2*Δ A, B | *MTL***a**/α, *sfl2*::*FRT*/*sfl2*::*FRT* | This study |
| SC5314*sfl2*Δ-Hr A, B | SC5314*sfl2*Δ A, B | *MTL***a**/α, *sfl2*::*FRT*/*sfl2*::*FRT*, *OP4/op4::OP4p-GFP-CaHygB* | This study |
| SC5314*sfl2*Δ+*SFL2* A, B | SC5314*sfl2*Δ A, B | *MTL***a**/α, *sfl2*::*FRT*/*sfl2*::*SFL2-CaSAT1* | This study |
| SC5314*sfl2*Δ+*SFL2*/*SFL2* A, B | SC5314*sfl2*Δ+*SFL2* A, B | *MTL***a**/α, *sfl2*:: *SFL2-CaHygB*/*sfl2*::*SFL2-CaSAT1* | This study |
| SC5314*sfl2*Δ*wor1*Δ A, B | SC5314*sfl2*Δ A, B | *MTL***a**/α, *sfl2*::*FRT*/*sfl2*::*FRT*, *wor1*::*FRT*/*wor1*::*FRT* | This study |
| SC5314*efg1*Δ A, B | SC5314 | *MTL***a**/α, *efg1*::*FRT*/*efg1*::*FRT* | This study |
| SC5314*efg1*Δ+*EFG1* A, B | SC5314*efg1*Δ A, B | *MTL***a**/α, *efg1*::*FRT*/*efg1*::*EFG1-CaSAT1* | This study |
| SC5314*efg1*ΔmCh A, B | SC5314*efg1*Δ A, B | *MTL***a**/α, *efg1*::*FRT*/*efg1*::*FRT*, *OP4/op4::OP4p-GFP-CaHygB* | This study |
| SC5314*sfl2*Δ*efg1*Δ A, B | SC5314*sfl2*Δ A, B | *MTL***a**/α, *efg1*::*FRT*/*efg1*::*FRT*, *sfl2*::*FRT*/*sfl2*::*FRT* | This study |
| SC5314*sfl2*Δ*efg1*ΔmCh A, B | SC5314*efg1*Δ*sfl2*Δ A, B | *MTL***a**/α, *efg1*::*FRT*/*efg1*::*FRT*, *sfl2*::*FRT*/*sfl2*::*FRT*, *OP4/op4::OP4p-GFP-CaHygB* | This study |
| P37039*SFL2*/*sfl2*Δ A, B | P37039 | *MTL***a**/α, *SFL2*/*sfl2*::*FRT* | This study |
| P37039*sfl2*Δ A, B | P37039*SFL2*/*sfl2*Δ A, B | *MTL***a**/α, *sfl2*::*FRT*/*sfl2*::*FRT* | This study |
| P37039*sfl2*Δ-Hr A, B | P37039*sfl2*Δ A, B | *MTL***a**/α, *sfl2*::*FRT*/*sfl2*::*FRT*, *OP4/op4::OP4p-GFP-CaHygB* | This study |
| P37039*sfl2*Δ+*SFL2* A, B | P37039*sfl2*Δ A, B | *MTL***a**/α, *sfl2*::*FRT*/*sfl2*::*SFL2-CaSAT1* | This study |
| P37039*sfl2*Δ+*SFL2*/*SFL2* A, B | P37039*sfl2*Δ+*SFL2* A, B | *MTL***a**/α, *sfl2*:: *SFL2-CaHygB*/*sfl2*::*SFL2-CaSAT1* | This study |
| P37039*sfl2*Δ*wor1*Δ A, B | P37039*sfl*Δ*2* A, B | *MTL***a**/α, *sfl2*::*FRT*/*sfl2*::*FRT*, *wor1*::*FRT*/*wor1*::*FRT* | This study |
| P37039*efg1*Δ A, B | P37039 | *MTL***a**/α, *efg1*::*FRT*/*efg1*::*FRT* | This study |
| P37039*efg1*Δ+*EFG1* A, B | P37039*efg1*Δ A, B | *MTL***a**/α, *efg1*::*FRT*/*efg1*::*EFG1-CaSAT1* | This study |
| P37039*efg1*ΔmCh A, B | P37039*efg1*Δ A, B | *MTL***a**/α, *efg1*::*FRT efg1*::*FRT*, *OP4/op4::OP4p-GFP-CaHygB* | This study |
| P37039*sfl2*Δ*efg1*Δ A, B | P37039*sfl2*Δ A, B | *MTL***a**/α, *efg1*::*FRT*/*efg1*::*FRT*, *sfl2*::*FRT*/*sfl2*::*FRT* | This study |
| P37039*sfl2*Δ*efg1*ΔmCh A, B | P37039*efg1*Δ*sfl2*Δ A, B | *MTL***a**/α, *efg1*::*FRT*/*efg1*::*FRT*, *sfl2*::*FRT*/*sfl2*::*FRT*, *OP4/op4::OP4p-GFP-CaHygB* | This study |
| SC5314*efg1*Δ+*EFG1* A, B | SC5314*efg1*Δ A, B | *MTL***a**/α, *efg1*::*FRT*/*efg1*::*EFG1-CaSAT1* | This study |
| P37039*efg1*Δ+*EFG1* A, B | P37039*efg1*Δ A, B | *MTL***a**/α, *efg1*::*FRT*/*efg1*:: *EFG1-CaSAT1* | This study |
| SC5314*sfl2*Δ+*SFL2* A, B | SC5314*sfl2*Δ A, B | *MTL***a**/α, *sfl2*::*FRT*/*sfl2*::*SFL2-CaSAT1* | This study |
| P37039*sfl2*Δ+*SFL2* A, B | P37039*sfl2*Δ A, B | *MTL***a**/α, *sfl2*::*FRT*/*sfl2*:: *SFL2-CaSAT1* | This study |
|  |  |  |  |

**REFERENCES**

1. Gillum AM, Tsay EY, Kirsch DR. 1984. Isolation of the Candida albicans gene for orotidine-5'-phosphate decarboxylase by complementation of S. cerevisiae ura3 and E. coli pyrF mutations. Mol Gen Genet 198:179-82.

2. Pujol C, Messer SA, Pfaller M, Soll DR. 2003. Drug resistance is not directly affected by mating type locus zygosity in Candida albicans. Antimicrob Agents Chemother 47:1207-12.

3. Solis NV, Park YN, Swidergall M, Daniels KJ, Filler SG, Soll DR. 2018. Candida albicans White-Opaque Switching Influences Virulence but Not Mating during Oropharyngeal Candidiasis. Infect Immun 86.

4. Park YN, Daniels KJ, Pujol C, Srikantha T, Soll DR. 2013. Candida albicans forms a specialized "sexual" as well as "pathogenic" biofilm. Eukaryot Cell 12:1120-31.
