# Supplementary figures and images for: Roles of the Transcription Factors Sfl2 and Efg1 in White-Opaque Switching in a/α Strains of Candida albicans
Source: mSphere. 2019 Apr 17;4(2):e00703-18. doi: 10.1128/mSphere.00703-18 (PMC6470211; doi:10.1128/mSphere.00703-18)

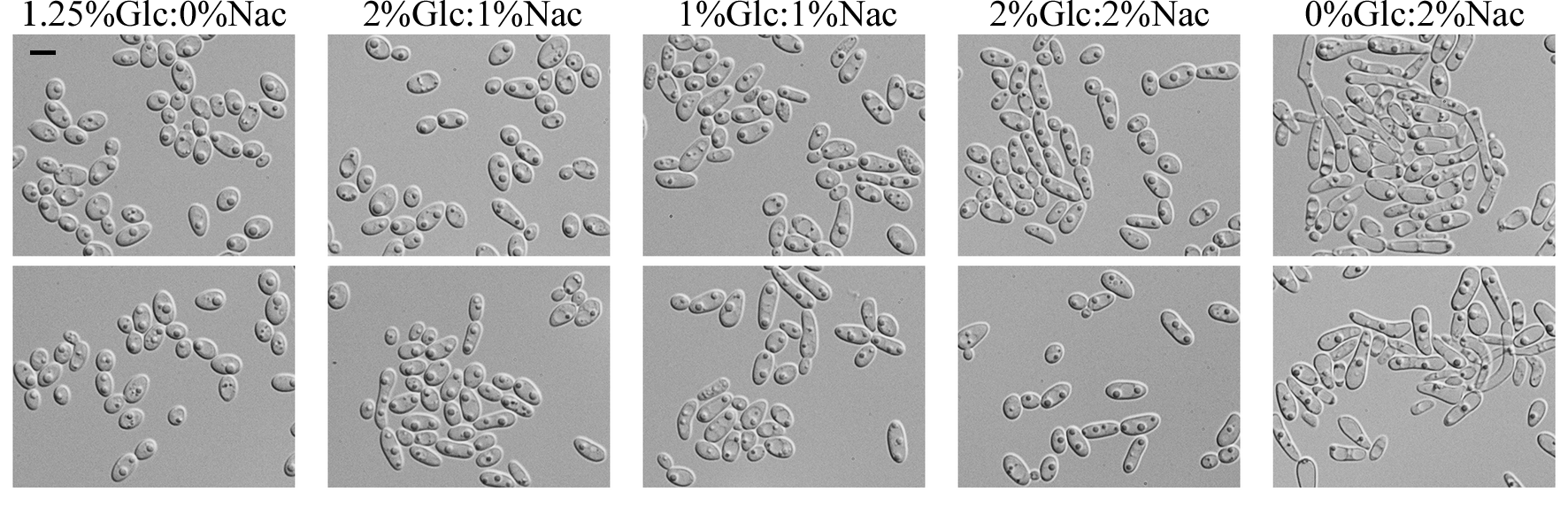

Supplement: FIG S1 [file mSphere.00703-18-sf001.tif]
