# Supplementary material for: Roles of the Transcription Factors Sfl2 and Efg1 in White-Opaque Switching in a/α Strains of Candida albicans
Source: mSphere. 2019 Apr 17;4(2):e00703-18. doi: 10.1128/mSphere.00703-18 (PMC6470211; doi:10.1128/mSphere.00703-18)
Supplement: TABLE S2 [file mSphere.00703-18-st002.docx]

**TABLE S2** Primers used in this study.

Primers used to generate mutants and to genotype *MTL*. Underlined sequences are the created restriction enzyme sites.

| **Primer** | **Sequence (5´ to 3´)** |
| --- | --- |
| EFG1-5´F | GAGAACAAAAGAAGGGCCCATTATTCATTGCAC |
| EFG1-5´R | TATACTCGAGTTATATTCTTGGTAGTCAAATAG |
| EFG1-3´F | ATATGCGGCCGCATTTGTGTACATCACCTTCTGCTTTC |
| EFG1-3´R | TTGTACCTTCCGCGGTAGACGCTTACTGCTTGC |
| EFG1-F | ATATGTCGACTTAATGTCAACGTATTCTATACCCTA |
| EFG1-R | TATAGGATCCAGAAAGCAGAAGGTGATGTACACAAATG |
| EFG1-3´F1 | ATATCTGCAGATTTGTGTACATCACCTTCTGCTTTC |
| mCherry-F | TATGTCGACATGGTTTCAAAAGGTGAAGAAG |
| mCherry-R | ATATGGATCCTTATTTATATAATTCATCCATACC |
| MTLa1F | TTGAAGCGTGAGAGGCAGGAG |
| MTLa1R | GATTAGGCTGTTTGTTCTTCTCG |
| MTLα2F | CATGAATTCACGTCTGGAGGCAC |
| MTLα2R | AAGCAGCCAACTCAGGTCAC |
| SFL2-5´F | GTTTGTTTTGGGCCCTTCCCCAATTTTGTGGG |
| SFL2-5´R | GCGTTCTTTTTCACTCGAGGATCACCAGG |
| SFL2-3´F | TCAGTTGCGGCCGCAGAAGAGAATAGTACCACTAGTAG |
| SFL2-3´R | AACACCGCGGGATGGGTTAACTATTAATATGCATCAG |
| SFL2-5´R1 | TATAGTCGACAATATAAGTATATGTGCATGTAATGTTAGAC |
| SFL2-F | ATATGTCGACAATGAGTAAGAAAAATCCTGGTG |
| SFL2-R | TAATACAATAAAGATCTATATATTATACAAC |
| SFL2-3´F1 | ATATCTGCAGAGTTTTATTGTATTAGAATTTTTCAATATAA |
| WOR1-5´F | GCCTATTGTTATGGTACCAACAGATTTCCACAT |
| WOR1-5´R | ATATGGGCCCCCTTAAATATATATAAGACAGATATTG |
| WOR1-3´F | ATATGCGGCCGCGTGGGTCTGTGTGTGAATTCGTG |
| WOR1-3´R | TATACCGCGGCAATATTACAATTCCCTTCATG |

Primers used for qRT-PCR.

| **Primer** | **Sequence (5´ to 3´)** |
| --- | --- |
| AHR1-f3 | CCAATCTAGAGCCAAATCCAGT |
| AHR1-r3 | TCCGACTCCCTTAAATCTTCTG |
| BRG1-f | TGCCCCATTACAATACGCCA |
| BRG1-r | ATGGCCTGGATGTTGATGCA |
| CDR3-f | TAGCAAGTGGAGAAAAGGAACC |
| CDR3-r | GGTGTACGCCAGTATTGTTGAA |
| CZF1-f2 | CGACTCAACAATATCCTGTCCA |
| CZF1-r2 | CAGCAGCAGAATACAAAGGTTG |
| CAG1-f | ATAGATGCTGATGTTGCTGGTG |
| CAG1-r | TCCCAGTACTCTTCAACCGTCT |
| EFG1-qf | TCAACAGGCTTTTCCTCAGC |
| EFG1-qr | TGTTGACCTGGTTGTCCTTG |
| MTLa1-qf | CAGAAACCCTTTTTCACGAGTC |
| MTLa1-qr | AGCTTCTTCTTCTTCCTGCTTG |
| MTLalpha2-qf | GGCTCTGGATAGTCCATTTTCA |
| MTLalpha2-qr | CCACCCTTCTAACACAAGCAAT |
| NDT80-f | AACAACAGCAGCAACAGCAG |
| NDT80-r | AATGTGGCTGCTGAGGTGTT |
| NRG1-f | AGCAGCTACTCCATTGTCACAA |
| NRG1-r | ATGGTATGGCTGAGGTTGTTGT |
| OP4-f | AAGAGCCGACAGTGTTATTGGT |
| OP4-r | TGGTAAGGGAGCTGGAAATAGA |
| RFG1-f | ATATTCAAGTGGGGCTGCCG |
| RFG1-r | TGAGAATGTGGTGGTGGAGG |
| SFL1-f | AAAGCCCCGACACCAGTAAA |
| SFL1-r | TGGCCTCCTTTTGTTGCTGT |
| SFL2-f | ATGTTGCATGATCCAGCGTT |
| SFL2-r | TCGGCAAATTCTTTTCCTGGA |
| SSN6-f | CTGGTATGATTGTTGCCGCAG |
| SSN6-r | AATCGCTGTTGTTGCTGCTG |
| TDH3-qf | ATGGGGTAAATCTGGTGTTGAC |
| TDH3-qr | AGCAGATGGAGCAGTGATGATA |
| TUP1-f | ACAGCAACAGCAGCAACAAC |
| TUP1-r | TTGGGTGGGGTTGACAATGT |
| UME6-f | TGATTTGCAACCACAGCCAC |
| UME6-r | TGGTTGGGATTGTGCTTGTTG |
| WOR1-f | GCGTAAATTTGGTCCCGACG |
| WOR1-r | CGCAAGCAACATTGGACCTG |
| WOR2-f | ACGTTGGCCCTCATCACATT |
| WOR2-r | AGCCAGTTCTTGTACGACGT |
| WOR3-f | AACGGTCGAGAAGATGGCAG |
| WOR3-r | TTCTGACCCACATCTGCGAC |
| WOR4-f | ATTCCACCCACAGACGTTGA |
| WOR4-r | GCTGGGTTGATTGCTGCATT |
| ZCF21-f | TGTCAACAGCAAATACAGCAGC |
| ZCF21-r | TGTTGATGCTGTTCCTGCTACT |
