# Supplementary material for: Roles of the Transcription Factors Sfl2 and Efg1 in White-Opaque Switching in a/α Strains of Candida albicans
Source: mSphere. 2019 Apr 17;4(2):e00703-18. doi: 10.1128/mSphere.00703-18 (PMC6470211; doi:10.1128/mSphere.00703-18)
Supplement: TABLE S3 [file mSphere.00703-18-st003.docx]

**TABLE S3** The **a**/α *sfl2*Δ*wor1*Δ double knock-out mutants did not switch to opaque cells. The total colony numbers were pooled from at least three independently performed experiments.

|  |  | 25°C, air | | 25°C, 5% CO2 | | 37°C, air | | 37°C, 5% CO2 | |
| --- | --- | --- | --- | --- | --- | --- | --- | --- | --- |
| Strains | Carbon source | Total col. no. | Frequency | Total col. no. | Frequency | Total col. no. | Frequency | Total col. no. | Frequency |
| SC5314*sfl2*Δ*wor1*Δ | Glucose | 3502 | < 2.9×10^-4^ | 3358 | < 3.0×10^-4^ | 3048 | < 3.3×10^-4^ | 3010 | < 3.3×10^-4^ |
|  | GlcNAc | 3006 | < 3.3×10^-4^ | 3142 | < 3.2×10^-4^ | 3508 | < 2.9×10^-4^ | 5262 | < 1.9×10^-4^ |
| P37039*sfl2*Δ*wor1*Δ | Glucose | 2798 | < 3.6×10^-4^ | 2784 | < 3.6×10^-4^ | 2630 | < 3.8×10^-4^ | 2532 | < 3.9×10^-4^ |
|  | GlcNAc | 2902 | < 3.4×10^-4^ | 2962 | < 3.4×10^-4^ | 2636 | < 3.8×10^-4^ | 4098 | < 2.4×10^-4^ |

Total col. no., total colony number.
