# Supplementary material for: Roles of the Transcription Factors Sfl2 and Efg1 in White-Opaque Switching in a/α Strains of Candida albicans
Source: mSphere. 2019 Apr 17;4(2):e00703-18. doi: 10.1128/mSphere.00703-18 (PMC6470211; doi:10.1128/mSphere.00703-18)
Supplement: TABLE S4 [file mSphere.00703-18-st004.docx]

**TABLE S4** White to opaque switching in different concentrations of glucose and GlcNAc, as well as in combinations of the two sugars. The agar cultures were incubated for 3 days at 37°C in 5% CO_2_, prior to analysis of colony and cell morphology. Opaque-sectored colonies were counted as opaque. The data represents the means ± standard deviations of two or more experiments.

|  | **White-to-opaque switching frequency (%)** | | | | | | | |
| --- | --- | --- | --- | --- | --- | --- | --- | --- |
| Strains | 0.1% Glc | 1% Glc | 1.25% Glc | 2% Glc  +  1% GlcNAc | 1% Glc  +  1% GlcNAc | 2% Glc  +  2% GlcNAc | 1% GlcNAc | 2% GlcNAc |
| SC5314 wt | 0 | 0 | 0 | 0 | 0 | 0 | 0.0 | 0.0 |
| SC5314*sfl2*Δ | 0 | 0 | 0 | 0 | 0 | 0 | 46.4±30.6 | 94.3±1.1 |
| SC5314*efg1*Δ | 100 | 0 | 0 | 0 ^a^ | 0 ^a^ | 0 ^a^ | 100.0 | 100.0 |
| SC5314*sfl2*Δ*efg1*Δ | 100 | 0 | 0 | 0 ^a^ | 0 ^a^ | 0 ^a^ | 100.0 | 100.0 |
| P37039 wt | 0 | 0 | 0 | 0 | 0 | 0 | 0.0 | 0.0 |
| P37039*sfl2*Δ | 0 | 0 | 0 | 0 | 0 | 0 | 77.8±13.2 | 98.0±1.4 |
| P37039*efg1*Δ | 100 | 0 | 0 | 0 ^a^ | 0 ^a^ | 0 ^a^ | 100.0 | 98.4±3.2 |
| P37039*sfl2*Δ*efg1*Δ | 100 | 0 | 0 | 0 ^a^ | 0 ^a^ | 0 ^a^ | 99.0±2.0 | 99.3±1.4 |

^a^: White colonies containing a mixture of yeast and opaque cells, varying in proportion (see Fig. S1). wt, wild type.
