# Supplementary material for: Roles of the Transcription Factors Sfl2 and Efg1 in White-Opaque Switching in a/α Strains of Candida albicans
Source: mSphere. 2019 Apr 17;4(2):e00703-18. doi: 10.1128/mSphere.00703-18 (PMC6470211; doi:10.1128/mSphere.00703-18)
Supplement: TABLE S5 [file mSphere.00703-18-st005.docx]

**Table S5** White-to-opaque switching frequency of the *SFL2* and *EFG1* complemented strains. The total colony numbers of at least two independently performed experiments were pooled.

| Strains |  | 25°C, air | | 25°C, 5% CO_2_ | | 37°C, air | | 37°C, 5% CO_2_ | |
| --- | --- | --- | --- | --- | --- | --- | --- | --- | --- |
|  | Carbon source | Total col. no. | Frequency (%) | Total col. no. | Frequency (%) | Total col. no. | Frequency (%) | Total col. no. | Frequency (%) |
| SC5314  *SFL2*/*SFL2* | Glucose | 1278 | 0 | 1298 | 0 | 1287 | 0 | 1441 | 0 |
|  | GlcNAc | 1458 | 0 | 1251 | 0 | 1135 | 0 | 1401 | 0 |
| SC5314  *sfl2*Δ/*sfl2*Δ | Glucose | 3797 | 0 | 2033 | 0 | 3015 | 0 | 2740 | 0 |
|  | GlcNAc | 2101 | 0.1±0.2 | 3701 | 4.9±5.7 | 3298 | 0.0±0.1 | 2544 | 93.8±5.8 |
| SC5314 *SFL2*/*sfl2*Δ | Glucose | 2001 | 0 | 2210 | 0 | 1965 | 0 | 2045 | 0 |
|  | GlcNAc | 2031 | 0 | 2110 | 5.3 ± 5.6 | 2214 | 0 | 1997 | 5.3 ± 1.2 |
| SC5314 *sfl2*Δ/*sfl2*Δ  + *SFL2* | Glucose | 2596 | 0 | 2717 | 0 | 2623 | 0 | 2656 | 0 |
|  | GlcNAc | 2449 | 0 | 2432 | 4.5 ± 0.7 | 2652 | 0 | 2471 | 11.7 ± 2.7 |
| SC5314 *sfl2*Δ/*sfl2*Δ  + *SFL2* | Glucose | 2074 | 0 | 2383 | 0 | 2274 | 0 | 2305 | 0 |
|  | GlcNAc | 2134 | 0 | 2195 | 0 | 2527 | 0 | 2471 | 0 |
|  |  |  |  |  |  |  |  |  |  |
|  |  |  |  |  |  |  |  |  |  |
| P37039  *SFL2*/*SFL2* | Glucose | 1464 | 0 | 1201 | 0 | 1299 | 0 | 1315 | 0 |
|  | GlcNAc | 1330 | 0 | 1178 | 0 | 1156 | 0 | 1370 | 0 |
| P37039  *sfl2*Δ/*sfl2*Δ | Glucose | 2618 | 0 | 1734 | 0 | 2038 | 0 | 2128 | 0 |
|  | GlcNAc | 1969 | 0 | 2837 | 1.9±3.6 | 1763 | 0 | 2243 | 99.6±0.6 |
| P37039 *SFL2*/*sfl2*Δ | Glucose | 1809 | 0 | 1611 | 0 | 1618 | 0 | 1599 | 0 |
|  | GlcNAc | 1601 | 0 | 1723 | 0.9 ± 0.9 | 1811 | 0 | 1728 | 9.3 ± 4.9 |
| P37039 *sfl2*Δ/*sfl2*Δ  + *SFL2* | Glucose | 2108 | 0 | 2309 | 0 | 2020 | 0 | 2213 | 0 |
|  | GlcNAc | 1934 | 0 | 2131 | 2.8 ± 0.7 | 2344 | 0 | 2002 | 8.7 ± 2.7 |
| P37039 *sfl2*Δ/*sfl2*Δ  + *SFL2* | Glucose | 1768 | 0 | 1828 | 0 | 1709 | 0 | 1771 | 0 |
|  | GlcNAc | 1690 | 0 | 1958 | 0 | 1847 | 0 | 1707 | 0 |
|  |  |  |  |  |  |  |  |  |  |
|  |  |  |  |  |  |  |  |  |  |
| SC5314  *efg1*Δ/*efg1*Δ  + *EFG1* | Glucose | 1161 | 0 | 1073 | 0 | 1166 | 0 | 1163 | 0 |
|  | GlcNAc | 1140 | 0 | 1097 | 0 | 1055 | 0 | 1107 | 0 |
| P37039 *efg1*Δ/*efg1*Δ  + *EFG1* | Glucose | 1229 | 0 | 1243 | 0 | 1077 | 0 | 1129 | 0 |
|  | GlcNAc | 1208 | 0 | 1215 | 0 | 979 | 0 | 970 | 0 |
|  |  |  |  |  |  |  |  |  |  |

Total col. no., total colony number.
